# Gamified Digital Mental Health Interventions for Young People: Scoping Review of Ethical Aspects During Development and Implementation

**DOI:** 10.2196/64488

**Published:** 2024-11-28

**Authors:** Wanda Spahl, Valeria Motta, Kate Woodcock, Giovanni Rubeis

**Affiliations:** 1 Division Biomedical and Public Health Ethics Karl Landsteiner University of Health Sciences Krems Austria; 2 School of Psychology University of Birmingham Birmingham United Kingdom; 3 The Centre for Developmental Science School of Psychology University of Birmingham Birmingham United Kingdom; 4 Institute for Mental Health School of Psychology University of Birmingham Birmingham United Kingdom

**Keywords:** ethics, digital health, eHealth, mobile health, mHealth, mental health, gamification, youth, young people, mobile phone

## Abstract

**Background:**

Young people are particularly at risk of developing mental health problems, a challenge exacerbated by the COVID-19 pandemic. Digital tools such as apps and chatbots show promise in providing accessible, cost-effective, and less stigmatized ways of strengthening their mental health. However, while these interventions offer benefits, they extend mental health measures beyond traditional therapeutic settings and relationships, which raises ethical concerns due to the absence of established guidelines and regulations. This is particularly notable for technologies incorporating serious gaming elements. In addition, adolescents are in a sensitive and at times vulnerable phase, which shows great potential for the effective use of preventive and sensitizing mental health measures. Considering the lack of an integration into existing mental health structures among many young users, ethical considerations become crucial.

**Objective:**

This scoping review aims to build a knowledge base on the ethical aspects of developing and implementing gamified digital mental health interventions for young people.

**Methods:**

We conducted a search on research articles and conference papers from 2015 to 2023 in English, German, and Spanish. We identified 1815 studies using a unique combination of keywords in the databases Scopus, Web of Science, MEDLINE, and PsycINFO. After removing duplicates (741/1816, 40.8%), we included a total of 38 publications in this review following a double screening process.

**Results:**

This review found that ethically relevant aspects were discussed with regard to (1) research ethics, (2) ethical principles (including privacy, accessibility, empowerment and autonomy, cultural and social sensitivity, and co-design), (3) vulnerable groups, and (4) social implications (including implementation using facilitators in specific social contexts, relationship with other therapeutic options, economic aspects, and social embeddedness of technologies).

**Conclusions:**

This scoping review identified a prevailing limited interpretation of “ethics” as research ethics across the included publications. It also shows a lack of discussion on the social embeddedness of technologies and that co-design is frequently viewed in instrumental terms and vulnerability is mostly addressed pragmatically. Through providing concrete examples of how mental health researchers and game designers thus far have addressed and mitigated ethical challenges in specific interventions, this review illustrates how ethical issues do or do not prompt diverse reflections, mitigation strategies, and actions. It advocates for ethics to be integrated as an ongoing practice throughout all stages of developing and implementing serious game elements in mental health interventions for young people.

## Introduction

### Background

Mental health is a major global concern that has been exacerbated by the COVID-19 pandemic [1]. The stage of life up to and including adolescence represents a window of both particular sensitivity and vulnerability but also opportunity with respect to mental health. Aversive experiences during this time can establish a lifetime trajectory of poor mental health, whereas a number of supportive factors can protect future mental health [2]. Young people today are often described as “digital natives” due to their familiarity with digital technology, having grown up with it [3]. Consequently, digital mental health interventions [4,5], including digital games [6], are particularly promising in reaching and supporting this population with accessible, cost-effective, and less stigmatized ways of strengthening their mental health [7].

While digital mental health interventions offer benefits, they extend mental health measures beyond traditional therapeutic settings and relationships and, thereby, raise specific ethical concerns [8,9]. Although studies and guidelines on the ethical aspects of digital mental health technologies have been published [10-12], there is a knowledge gap regarding young people. Guidelines and regulations are still needed for this group [13]. A rare exception is one scoping review on the ethical aspects of digital mental health for young people that included studies up to October 2020. It found ethical potential related to accessibility, therapy facilitation and prevention, empowerment, and high acceptability. Risks concerned privacy, patient mistrust, stigma, access inequalities, cross-cultural differences, clinical validation, ethical and legal guidance, and consent [14]. Considering the rapid technological advancements, new insights are likely available now. In addition, the aforementioned review did not explicitly focus on serious games, namely, digital interactive tools designed to address mental health issues through engaging gameplay. Thus far, the literature on the ethics of serious games [15-17] has largely evolved separately from the literature on mental health interventions for young people.

In light of this gap in the ethics literature, this scoping review showed ethical aspects of gamified digital mental health interventions for young people aged between 10 and 25 years. Such interventions have been designed for a broad spectrum of mental health needs, ranging from preventive measures for the general youth population to treatment for mild and severe conditions [18]. We define mental health broadly to include specific mental health diagnoses as well as more general well-being related to emotional regulation and feelings of connectedness and belonging. In this review, digital interventions encompass those designed for interactive use on computers and mobile devices and in extended realities such as augmented and virtual reality. This review included interventions self-identified as games or incorporating gamified elements without focusing on specific game mechanics.

While we focused on specific predefined ethical principles and issues, such as autonomy, empowerment, privacy, and equity, we also explored additional ethical considerations that emerged from the discussed papers. This review conceptualized ethical aspects as multifaceted concerns that arise throughout all stages of intervention development and implementation. These extend beyond research ethics and institutional approvals to also encompass game design decisions and broader considerations, such as environmental impact, economic factors related to funding, and the roles of facilitators such as teachers and therapists.

### Objectives

This review identified the various ethical considerations discussed by developers and researchers when describing specific interventions for young people that include gameplay elements. We asked the following research questions (RQs):

- What are ethical aspects of gamified digital mental health interventions for young people? (RQ 1)
- What needs to be considered in the development of gamified digital mental health interventions for adolescents to mitigate ethical challenges? (sub-RQ 1)
- What ethical aspects does the literature identify with regard to vulnerable groups and who is identified as a vulnerable group (eg, specific diagnoses and social markers)? (sub-RQ 2)
- What are relevant social implications (eg, public or private funding, school-based or home-based environment, and regulatory frameworks)? (sub-RQ 3)

This review is part of the larger Horizon Europe–funded project “ASP*belong*” (2023-2027 [19]). The project aims to develop Augmented Social Play, a smartphone-based group psychotherapeutic intervention that enhances adolescent mental health by fostering real-world connections and a sense of belonging.

## Methods

Following established scoping review guidelines ([20]; Multimedia Appendix 1 [21]), the selection process for the literature search followed 4 phases: identification, screening, eligibility, and inclusion. Moreover, the search was complemented by asking all team members of the interdisciplinary Horizon 2022 project ASP*belong*, as well as the participants of a 2023 workshop on prosocial games in extended realities at the 22nd International Conference on Mobile and Ubiquitous Multimedia [22], for additional publications meeting our inclusion criteria—resulting in the inclusion of one more publication.

### Literature Search

To identify studies for our review, we used the scientific databases Scopus, Web of Science, MEDLINE, and PsycINFO. These databases allowed for searches across the different disciplines relevant to this scoping review’s topic, including ethics, psychology, computer sciences, and design research. To answer our RQs, we aimed to find existing research on (1) ethical aspects of (2) gamified (3) digital (4) mental health interventions for (5) adolescents. Each part (1-5) was operationalized using a string of search terms. The search terms were combined using the Boolean operators OR (within search strings) and AND (across search strings), adapting the operators and syntax for different databases as necessary (for the full list of search terms and the search strings for each database, see Multimedia Appendix 2).

To identify search terms for strings 2 to 5, we oriented ourselves using existing reviews on similar topics in high-quality journals [23-25], modifying them to this review’s focus and requirements. For the identification of search terms related to the ethical aspects (search string 1), existing reviews provided only moderate assistance. In the field of ethics, scoping reviews are still developing as a methodology. The few existing reviews on ethical aspects of digital mental health interventions have either omitted the outcome of ethics to avoid excessively narrowing the search [26] or used solely the search term “ethics” or combinations thereof, such as “bioethical issues,” “ethical analysis,” and “ethical review” [14]. Neither of these search strategies proved viable for our review. Initial searches yielded insufficient results when combining search strings 2 to 5 with “ethics” or related combinations (35 results in Scopus compared to 755 results retrieved using the final search string). Conversely, the number of results increased significantly when no ethics-related search string was included (1245 results in Scopus).

In response to these considerations, we created a new search string for ethical aspects that included not only the search term “ethics” or combinations thereof but also concrete examples of ethical principles and issues. The search terms were inspired by a briefing note on the role of technology in mental health care by the Nuffield Council on Bioethics [11] and on search terms used in preceding reviews on ethical aspects of digital health technologies [14,27,28]. The selection process involved collaborative brainstorming within the author team and was led by the ethics experts in our authorship team (GR and WS). The final search terms related to ethical aspects included (1) explicitly ethics-related search terms, (2) more general search terms for challenges and advantages, (3) concrete ethical principles and issues, and (4) a focus on access and equity (Textbox 1).

**Search group for identifying ethical aspects.**

**Theme and search terms**

- Explicitly ethics-related search terms: *ethics, ethical, moral, value, ELSI,* and *ELSA*
- More general search terms for challenges and advantages: *risk, benefit, potential,* and *challenge*
- Concrete ethical principles and issues: *autonomy, empowerment, privacy, confidentiality, trust, consent, stigma, responsibility, regulatory framework,* and *safety*
- Focus on access and equity: *accessibility, equity, inequity, equality, inequality, bias, digital literacy, socioeconomic, social determinant,* and *exclusion*

### Eligibility Criteria

We included research articles and conference papers in English, German, and Spanish (languages spoken by the authors) that were published between 2015 and 2023. The rationale for excluding publications before 2015 was the rapid evolvement of digital health technologies that makes it difficult to compare newer digital interventions to older ones (eg, showing a patient a video as a digital intervention before 2015 vs the newest augmented reality technologies). Publications considered for inclusion addressed ethical aspects of gamified digital mental health interventions for young people (aged 10-25 years). Textbox 2 provides a detailed list of the inclusion and exclusion criteria.

**Inclusion and exclusion criteria.**

**Inclusion criteria**

- Period: papers published between 2015 and 2023
- Study types: Research articles and conference papers (plus conference abstracts if titles or abstracts focused on ethics to contact the authors for further information)
- Language: English, German, and Spanish
- Population: young people (aged 10-25 years; note for the scanning process: include studies with, eg, age ranges of 8-12 or 18-30 years)
- Intervention: the intervention was gamified, including interventions with a gamified aspect; the intervention was digital, including extended realities (augmented and virtual reality) and apps and other interactive content that can be used on mobile and other devices (ie, smartphones and computers); the intervention aimed at increasing, stabilizing, or informing about mental health, including mental illness prevention. Mental health is understood in a broad sense, including feelings of well-being, belonging, social connection, and relatedness; psychotherapeutic and nonpsychotherapeutic interventions
- Outcome: the publication addressed ethical challenges and advantages when designing and implementing gamified digital mental health interventions.

**Exclusion criteria**

- Period: papers published before 2015 and after 2023
- Study types: systematic reviews or reviews, opinion papers, editorials, special issue introductions, doctoral theses, workshops, protocols, and textbooks
- Language: other languages
- Population: people aged <10 years and >25 years; parenting interventions (eg, interventions designed for parents and interviews with or surveys on parents); interventions that were tested with students without being designed for young people
- Intervention: the publication studied the harmfulness of excessive media consumption; the intervention was not a mental health intervention but aimed at, for example, improved physical health or increasing learning motivation; the publication was not based on insights from an existing intervention, including prototype development, implementation, and user experiences (eg, broad and abstract introductory publications such as “Addressing children’s mental health issues in the 21st century”); the intervention was a digital tool just to screen for mental health status (ie, assessment tool); the intervention was tested with students without being designed for young people; the intervention was aimed at screening the current mental health state (eg, at the start of conventional therapy)
- Outcome: the publication only mentioned the ethics committee’s vote or briefly described some aspects of research ethics (eg, obtaining parental consent) without other discussions of ethical aspects; the publication only reported on ethically relevant decisions without discussing them.

### Screening

Duplicates were removed from the collated papers for screening (using the review software Covidence [Veritas Health Innovation] and by hand). In total, 2 independent reviewers screened the remaining papers, regularly discussing inconsistencies and uncertainties in decisions (title and abstract screening: WS as first reviewer and 3 research assistants as second reviewers; they worked at the University of Birmingham during a work placement year between the second and final years of their bachelor’s degrees at other universities and received training and regular check-ins; full-text screening: VM and WS). During title and abstract screening, we did not attend to the outcome inclusion criteria, which addressed ethical aspects. While the terms in the search group for identifying ethical aspects (Textbox 1) aimed to ensure the ethical relevance of the search hits, when jointly screening the titles and abstracts of the first papers (approximately 50 titles and abstracts assessed together by VM and WS), we realized that a full-text screening was necessary to determine the relevance of the publication to our RQs. We attuned our eligibility criteria when proceeding with abstract and title screening, adding further exclusion criteria (ie, interventions tested with students without being designed for young people and interventions aimed at screening mental health status). Early screened papers were reassessed to align with the revised criteria.

During full-text screening, the focus was on the publications’ discussion of ethical aspects. We remained oriented to our predetermined topics, aligning with the search terms related to ethical aspects (Textbox 1). In addition, we worked inductively, being mindful to the discussion of other ethical aspects.

We excluded publications that did not report on any ethically relevant reflections or discussions. Papers that only mentioned the ethics committee’s vote or some aspects of research ethics in a brief way (eg, stating that parental consent was obtained when involving minors in intervention effectiveness measurement) were also excluded. We argue that simply including the reference to an obtained ethics committee vote and the brief addressing of consent procedures does not constitute ethical reflection but can best be understood as good scientific practice or as an appeal to authority. Moreover, we excluded publications that mentioned ethically relevant decisions (eg, design decisions on having avatars of different genders) but did not provide any reflections or further discussions of these decisions. Inconsistencies were jointly resolved in dedicated meetings between VM and WS.

### Data Extraction and Analysis

Our approach to data analysis can be best described as an abductive approach [29] to ethics, being both theory informed and empirically oriented. This approach did not adhere to a rigid, predefined concept of ethics. Instead, we intentionally maintained an open and flexible definition of ethics. We drew upon ethical considerations mentioned in existing research and guidelines on digital mental health interventions, as well as our own experiences. This flexibility facilitated the exploration of unanticipated ethical aspects in an inductive manner. We began analyzing data during the full-text screening phase as this process helped refine our understanding of what ethical aspects may or may not entail. Thereafter, data analysis followed several steps.

First, we created a template for extracting data from the 38 included publications with the easy-to-adapt tool from the review software Covidence (“data extraction template 2,” which is designed for customized reviews, such as scoping reviews). This template included categories for general information on the paper (title, authors, publication year, and outlet), the characteristics of the study (discipline, aim of the study, and method), the intervention (country, intervention funding sources, target population, intervention name, media, type of gamification, co-design elements, mental health understanding, and overview of the ethical aspects addressed in the publication), and ethical aspects (“research ethics (detailed considerations),” “ethical principle privacy,” “ethical principle: other,” “value conflicts,” “vulnerable groups,” “social implications,” and “other ethical aspects”). Information on ethical aspects was inserted within open-ended fields in the form of direct quotes preceded by a short summary of a few words. [Author] performed data extraction as a single reviewer, including a simple check on all the extracted data after completing extraction in Covidence plus harmonizing the data and cleaning them from mistakes after conversion to Microsoft Excel.

Second, we refined the list of ethical aspects in our data extraction template during data extraction in an inductive manner adding 4 subcategories that were mentioned across a considerable number of studies (ie, “ethical principle: accessibility,” “value conflicts: entertainment vs. psychological/educational value,” “other ethical aspects: avatar diversity,” and “other ethical aspects: value of co-design”). Third, we clustered the insights on each ethical aspect into meaningful topics based on our short topical summaries of each extracted quote. This step also involved the formation of new subaspects as they emerged from the data (eg, “cultural and social sensitivity” emerged as a new subaspect of “ethical principles”; see the Results section). Fourth, we analyzed the publications’ discussions of each ethical aspect and subaspect identified as a result based on the direct quotes. We described similarities across the studies as well as special or unique ethical insights (for a table with all the direct quotes and summaries extracted, see Multimedia Appendix 3 [30-66]).

## Results

### Overview

**Figure 1:**
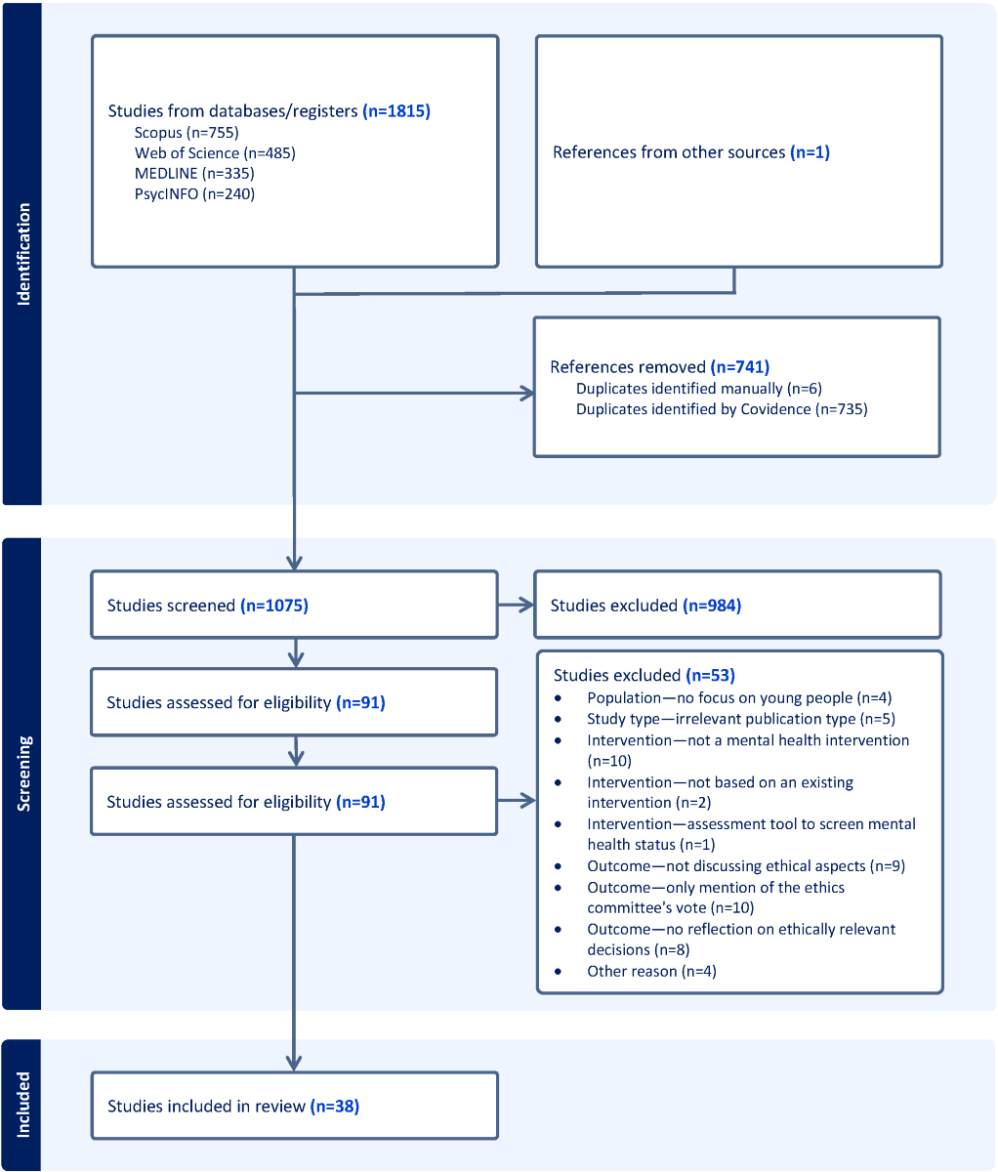
PRISMA (Preferred Reporting Items for Systematic Reviews and Meta-Analyses) flow diagram.

A total of 1075 studies were screened after removal of duplicates (741/1816, 40.8%). Of the 91 publications that were reviewed in full text, we excluded 22 (24%) because the described interventions did not match our inclusion criteria, 27 (30%) because they did not include ethically relevant outcomes, and 4 (4%) based on other reasons (Figure 1). In the following, we outline the main characteristics of the publications included, followed by a discussion of the ethical aspects addressed by them.

### Main Characteristics of the Included Publications

The 38 publications included in this review addressed 32 interventions, whereby 2 (5%) of the papers discussed 3 (9%) of the interventions each (for a list of each study’s key characteristics, see Multimedia Appendix 4 [30-66]). Table 1 shows a summarized overview of the studies’ key characteristics. There was a leaning toward more publications in later years. The distribution of studies showed a focus on the Global North, with most interventions developed in Europe (most of them in the Netherlands and Spain, with 3/32, 9% of the interventions each) followed by North America (7/32, 22% in the United States and 2/32, 6% in Canada) and Oceania (4/32, 12% in Australia; 3/32, 9% in New Zealand; and 1/32, 3% as a collaboration between the 2 countries).

**Table 1:** Overview of the included publications’ key characteristics (N=38).

		Studies, n (%)^a^
**Year of publication**		
	Between 2015 and 2017	8 (21)
	Between 2018 and 2020	10 (26)
	Between 2021 and 2023	20 (53)
**Continent**		
	Europe	15 (39)
	North America	9 (24)
	Oceania	8 (21)
	Asia	4 (11)
	Africa	1 (3)
	South America	1 (3)
**Authors’ disciplines**		
	Interdisciplinary	13 (34)
	Psychology	11 (29)
	Human-computer interaction	7 (18)
	Medicine	3 (8)
	Education	2 (5)
**Aim of the study**		
	Understand user experiences: mixed methods	12 (32)
	Present intervention design	9 (24)
	Measure intervention impact	6 (16)
	Understand user experiences: qualitative methods	6 (16)
	Understand user experiences: quantitative methods	3 (8)
	Other	3 (8)
**Intervention funding source**		
	Not mentioned	18 (47)
	Nongovernmental organization	5 (13)
	University	5 (13)
	State institution	5 (13)
	Mixed funding	4 (11)
	Research grant	1 (3)
**Target population**		
	Vulnerable groups	15 (39)
	Specific age group	8 (21)
	Young people in school	6 (16)
	Other	4 (11)
	University students	3 (8)
	Young people in face-to-face therapy	2 (5)
**Digital media**		
	Mobile app	18 (47)
	Web-based video game	11 (29)
	Other	9 (24)
**Co-design elements**		
	Not mentioned	17 (45)
	Co-design approach	12 (32)
	Feedback from young people during development	9 (24)
**Mental health issue addressed**		
	Preventive	11 (29)
	Anxiety or depression	9 (24)
	Loneliness and belonging	5 (13)
	Other	5 (13)
	Social skills	4 (11)
	Mental health–related issue	4 (11)
**Therapeutic approach**		
	Other	22 (58)
	Cognitive behavioral therapy	10 (26)
	Socioemotional learning	4 (11)
	Behavioral activation theory	1 (3)
	Stress coping theory	1 (3)

^a^Some deviation in the cumulative sum due to decimal rounding.

More than half (21/38, 55%) of the studies aimed to understand user experiences, mostly applying mixed methods designs, such as combining surveys among young people with qualitative methods, or solely qualitative methods. Among the qualitative methods used were interviews with young people, teachers, school-based health providers, therapists, and parents, as well as focus groups and co-design workshops. Nearly half (18/38, 47%) of the publications did not indicate the funding source of the intervention, which is interesting from an ethical point of view because different donors can be expected to have different interests and influence certain decisions of intervention design. One-third of the publications (15/38, 39%) addressed interventions for what we interpreted as vulnerable groups specifically targeting young people with specific mental, physical, or social needs rather than interventions based on age or educational level.

In terms of digital media, nearly half (18/38, 47%) of the publications addressed interventions created as mobile apps, followed by web-based video games. There was a noticeable trend of increasing mobile app development over time, with a median publication year of 2022, compared to web-based video games, which had a median publication year of 2019. In addition, 9% (3/32) of the interventions incorporated biofeedback technologies; 3% (1/32) used automated, interactive SMS text messaging; 3% (1/32) were collaborative augmented reality apps; 3% (1/32) were designed as a computer-based game; and another (1/32, 3%) was designed for PC, website, and mobile app platforms. Notably, 3% (1/38) of the publications defined the intervention as comprising not only a mobile app but also in-person introductory lessons led by a researcher as well as a debriefing session facilitated by a person with lived experience of depression [30]. The integration of these face-to-face interactions alongside the digital components provides a holistic approach and can add depth to the digital experiences of young people.

The most frequently used therapeutic approaches were cognitive behavioral therapy (12/38, 30% of the publications) and socioemotional learning (5/38, 13% of the publications). Others included behavioral activation theory (1/38, 3%), stress coping theory (1/38, 3%), and a strengths-based positive psychology framework (1/38, 3%). While few (3/38, 8%) reported a mix of several approaches, a considerable number (15/38, 39%) of the publications did not indicate following one specific approach, remaining vague on the mental health framework informing the intervention.

Finally, nearly all the publications cited previous studies and policy guidelines on ethically relevant advantages of gamified digital mental health technologies for young people in their introductions or conclusions. These advantages typically included providing cost-effective treatment to better meet mental health needs, particularly in health care systems with limited access to care; increasing engagement with therapeutic content by leveraging young people’s affinity for games and new digital technologies; and reaching underserved populations, such as economically disadvantaged youth, adolescents in low- and middle-income countries, and individuals fearing stigma, through affordable and widely available digital technologies, especially smartphones.

### Detailed Considerations on Research Ethics

A total of 26% (10/38) of the publications provided detailed discussions on research ethics. In total, 20% (2/10) of these publications exhibited careful approaches to engaging with Indigenous youth in Canada [31] and New Zealand [32]. They reflected on their own positions by acknowledging the Western and Indigenous backgrounds of the author teams. In addition, they shared their experiences of working with Indigenous communities in previous projects and emphasized close collaboration with Indigenous communities from the outset of study development. For example, the study in Canada formed an Indigenous committee with community leaders from various areas with hourly compensation, which jointly devised recruitment strategies and study design. The Indigenous committee’s recommendations included qualitative interview data on adult community members’ view on mental health apps, culturally cointerpreting findings “within a cultural lens” [31], and planning to store data at the Indigenous community site in the course of “a post-study action plan” [31]. Moreover, the study in New Zealand adopted the Indigenous kaupapa Maori methodology. Instead of a focus on individual change for improved mental health common to Western psychological models, it emphasized connections to extended family, past generations, tribal identity, the environment, and spiritual and physical well-being [32].

Other detailed reflections on research ethics addressed sensitive recruitment strategies [33] (see also the Vulnerable Groups section); informed consent [34-37], which was highlighted as an iterative process (eg, providing ongoing study information and ensuring attentiveness during data collection, including active teacher involvement) in the study by Høiseth et al [34]; and specific financial compensations for study participants (eg, providing refreshments for and giving away smartphones to adolescents participating in co-design activities in sub-Saharan Africa in the study by Pozuelo et al [37]). Furthermore, there were discussions on implementing additional safeguards for working sensitively with vulnerable groups (eg, recruiting people with psychosis through early psychosis teams, researchers carefully screening for acute symptoms, and maintaining regular contact with participants via SMS text message or calls to support with technical issues and other concerns in the study by Lim et al [38]). Finally, 3% (1/38) of the publications addressed the power imbalance between researchers and vulnerable populations as well as the sensitive handling of data when collaborating with adolescents through a nongovernmental organization (eg, recordings uploaded by nongovernmental organization members with permission for viewing but not for downloading for researchers in the study by Sockolow et al [39]).

### Ethical Principles

We now focus on how the publications implicitly and explicitly addressed the ethical principles of privacy, accessibility, empowerment and autonomy, and cultural and social sensitivity. Strengthening the principle of transparency at the meta level of communicating design decisions, one publication stated that “serious games for mental health are seldom described in depth and there is little research to elucidate components of serious games that might be useful or appealing” [40].

#### Privacy

Approximately one-third (12/38, 32%) of the publications addressed privacy considerations [31,35-37,40-47]. These were often raised as concerns by young people themselves who wanted to know how their data were used and protected and often preferred not to give their personal data [31,40,45]. For example, one proposed solution to address privacy concerns was to customize privacy settings in a way that allowed students to decide whether to upload a profile picture and share personal information within the app [42]. In an app used by adolescents in sub-Saharan Africa, where some individuals shared smartphones within households, the introduction of a personal unlocking code ensured confidentiality and contributed to establishing a “safe space” [37]. On a regulative level, one publication addressed adaptations to comply with the European Regulation on Data Protection [43].

Moreover, data collection during gameplay was discussed in relation to privacy safeguards. One publication addressed maintaining anonymity (children were assigned random animal “code names” for log-in) while categorizing players’ actions as “selfish,” “neutral,” or “cooperative” [36]. These categorizations were used solely for game analysis and were kept from the children to avoid causing any negative feelings. In another intervention, players chose their own personal strengths, with pre- and postmeasurement data collected via a deidentified code [47].

Furthermore, the publications addressed how to navigate conflicts between privacy and other values. A home video game for young children during the pandemic raised questions about spontaneous parental involvement [44]. While unintended benefits included “parents observing more closely their child’s emotional processing, reinforcing new skills, being actively involved after the sessions, and reflecting on their parenting role” [44], parent involvement also carried the risk of misconstruing the purpose of activities, sometimes even using them as punishment for their children [44]. Another study thematized the risk of clear communication on privacy settings disengaging users and aimed at balancing one with the other [45].

In total, 8% (3/38) of the publications discussed a conflict between maintaining confidentiality and privacy and ensuring user safety. Different target groups may require a different weighting of these values and should ideally be consulted on this [40]. In the case of a mood self-management app, young users expressed a preference for retaining control over seeking help. They suggested using a visible icon, such as heart-shaped hands, to represent a “safe space to chat” [45] rather than receiving notifications for immediate emergency assistance. In addition, they wished not to share personal details such as names, email addresses, and music preferences on the app to make sure they would not be put in contact with professional support services and avoid judgment from other users [45]. In contrast, in the context of an intervention aimed at suicide prevention, it was decided to send direct notifications in the case of concerning input, framed by openly communicated safety protocols and repeated information on further counseling options [48].

Beyond game design, the publications addressed the clear communication of privacy rights, for example, including a privacy policy in easy language within an app [46] or stressing the “importance of training both staff and students to...to ensure that they understand their rights to confidentiality and data privacy” [41] in the school context.

#### Accessibility

A total of 24% (9/38) of the studies addressed a variety of accessibility issues. One focus was on adaptations for vulnerable groups, such as socioeconomically disadvantaged adolescents from ethnic minority groups [39], Indigenous youth [31], and children with a diagnosis of autism spectrum disorder [49]. For example, it was recommended to use audio voice-overs for low-literacy populations [37]. In another study, an interviewed therapist expressed concerns about using text-based responses with children with anxiety, noting that one patient felt pressured to spell and punctuate perfectly, which increased anxiety. To address this issue, the authors instead recommended providing multiple-choice responses or emoticons [50]. A study from Lebanon highlighted the issue of the digital divide in the context of displaced youth, with some individuals lacking access to devices and facing unstable internet connections [51].

More generally, the term *accessibility* was used to describe certain design elements, such as operable and navigable functions, understandable text and storylines, and robustness and reliability [40], or a mechanism to encourage help-seeking behavior and reduce dropout rates [52]. In addition, discussions also covered accessibility issues in terms of technological aspects, including device availability [40] and the compatibility of the interventions with various types of devices, with a focus on improving access by ensuring functionality across both desktop computers and mobile devices [42,53]. This was achieved through strategies such as using the Unity3D game engine and reducing server workload to enhance response time [53]. One publication proposed to address limited storage space and unstable internet connections through “a low-storage app” [37] and exploring features that enabled offline access once the app was downloaded [37].

#### Empowerment and Autonomy

Some publications (4/38, 11%) addressed the ethical principles of empowerment and autonomy, foregrounding, for example, user-controlled choices as important [46]. One study found that users felt empowered by customization options such as preference settings. This allowed them to choose between plot options and select which information the app tracked [45]. Another study addressed how certain elements of serious games fostered autonomy through in-game activities such as exploring unfamiliar places; playing with another identity; customizing their own character; and providing perceivable and understandable information, an operable interface that allowed for pauses and the repetition of levels [40], technical robustness and reliability, and encouragement of self-management and feelings of nonjudgment as well as the simulation of real life [40]. Finally, one intervention aimed at neurodivergent children discussed autonomy as facilitated among therapists, parents, and children, for example, through collaboratively choosing in-app goals [54].

#### Cultural and Social Sensitivity

The studies addressed the need to adapt interventions to the target group’s cultural and social context. One recurring aspect, often highlighted by young people themselves [41], was character and avatar diversity [33,43], ranging from limited choices between male and female avatars [53] to a broad range of customization options, which ensured a cast of culturally relatable characters also with regard to ages, body shapes, social classes, and common names [31]. Young people’s own ethnicity and socioeconomic situation received particular reflection in two of the publications (2/38, 5%), which reported on qualitative insights from co-design sessions with adolescents from urban and socioeconomically disadvantaged communities in the United States [39] and with South African and Ugandan youth [37]. Both interventions adapted characters’ ethnicity (eg, the initial suggestion of characters in anime style replaced with a portrayal of adolescents’ body shapes and ethnicities deemed more appropriate by adolescent co-designers in the study by Sockolow et al [39]) and game esthetics (eg, esthetics informed by photographs of schools and nearby areas, as well as by the media preferences of the participants, in the study by Pozuelo et al [37]) to adolescents’ lived realities. In addition, the study by Sockolow et al [39] described adaptations with regard to language (using adolescents’ speech analysis) and nonplayer characters. “[C]haracter types that the adolescents often mentioned as supports or challenges as they made important decisions in their lives” [39], namely, the “trusted aunt,” the “good friend,” and the “jealous girlfriend,” were included [39]. Moreover, adolescents’ wish to address problems common to their daily lives, such as alcohol and cannabis consumption and teenage pregnancy, and solutions were translated into game design through “a set of interactive vignettes located in age-appropriate settings (for example, at school, home, playground, etc.)” [37].

Sensitivity toward young people’s social situation was also shown in considering the school context for an intervention set in a classroom via school-based workshops with adolescents [55], as well as the context of the COVID-19 pandemic marked by the fear of illness and the experience of isolation [44]. In addition, sensitivity toward country-specific cultural practices can inform design practices. One intervention adapted planned self-help goals to the strong reliance on authority figures in Indian schools and stressed the importance of incorporating local languages [46].

#### Co-Design

Around one quarter (9/38, 24%) of the studies highlighted the value of co-design approaches [32-34,39,41,47,52,55-57]. In general, co-design approaches hold ethical value as they involve the target group from the outset of intervention development and can, thereby, better address their needs and mitigate potential pitfalls. Studies understood co-design as a valuable process for aligning interventions with the needs and preferences of users [57], involving vulnerable target audiences such as minority groups [32,56], and obtaining firsthand insights into issues such as school refusal viewing youth as experts [34]. Often, the studies focused on the increase in engagement and enhancing effectiveness [32,47,52,55,56] in rather instrumental terms. For instance, one study justified youth involvement as a strategy to design games as “relevant, appealing, and optimally engaging to their target audience, increasing the probability that they will also be shared with family and friends” [52].

Furthermore, two of the publications (2/38, 5%) reflected on making the co-design process feasible as an iterative process over the entire development period [55,56], for example, aiming to involve enthusiastic young people over a longer period [56]. Another study illustrated the creativity inherent to applying co-design processes, for example, through using “active and spontaneous role play to elicit dialogue for script development” [39].

In addition, 13% (5/38) of the publications localized co-design not only as a practice targeted at user involvement but also involving multiple stakeholders and their knowledges [41,47,55,57]. For example, an opioid misuse prevention intervention engaged adolescents with and without misuse experiences, researchers on the topic, medical providers, and school representatives to reflect “their voices and perspectives for the greatest impact and reach” [41]. One intervention addressing the topic of young people living with parents with mental illness emphasized “multiprofessional co-development” [55] between adults with this experience and adolescents, as well as playwrights, game developers, computer scientists, and psychologists [55]. Similarly, another publication explicitly framed “a robust co-design framework that involved children, parents, teachers, clinicians, academics, and technical experts in prototype design, development, and evaluation via rapid user-testing” [57] as a strength of the intervention. Co-design was also understood as an ongoing working collaboration between game developers and psychology or education researchers [33,47].

#### Psychological and Educational Value

Nearly a quarter (8/38, 21%) of the publications addressed how to align the pedagogical goals of serious games targeting mental health with entertainment logics. Three of these studies (3/8, 38%) merely mentioned this conflict as a general challenge in gamified intervention development [36,52,67], for example, referring to the “critique of educational games being a ‘chocolate covered broccoli’” [36]. The other studies included reflections on how they practically navigated these values, foregrounding the value of interdisciplinary collaboration between mental health or education experts and game designers involved in project development [47,56,58]. One publication delineated an iterative process used by the development team for selecting therapeutic elements and their gamification, which was informed by young people acting as co-designers. It involved selecting the most beneficial psychological principles in a process of prioritization and feasibility reflection aimed at obtaining the “best ‘bang for our buck’” [56]. Critiquing conventional gamification approaches, the publications emphasized targeting players’ intrinsic motivations [45,58].

Moreover, the time- and energy-consuming practices of interdisciplinary collaboration among team members were expected to bring about “an exciting future...for games in the field of mental and emotional health” [58]. Another study on an in-therapy intervention showed how navigating psychological or educational and entertainment values depends on the previous experiences of users; an interviewed therapist cautioned against using the intervention for regular game players and the risk of adolescents using the intervention for entertainment only [50].

Finally, 2 of the publications (2/38, 5%) highlighted the potential of mitigating possible negative effects on mental health through game design. One publication emphasized young people’s worries about improper design, “such as when wording was explicitly directive rather than facilitating autonomous use” [45], worsening user well-being. Another publication used calming visuals and audio to counter bad mood effects from the game’s psychological or educational components [59].

### Vulnerable Groups

Targeting vulnerable groups generally raises distinct ethical considerations. Interventions were designed to address vulnerability across various factors, including socioeconomic background [39], existing mental [38,40,57] or physical [60] health conditions in young people or their parents, disabilities [49,54], belonging to an ethnic or gender minority group [61], indigeneity [31,32,56], the experience of forced displacement [51], and other factors such as school refusal [34].

Some studies (5/38, 13%) addressed representation and inclusivity concerns essential for meeting the needs of diverse populations [41,44,56,61,62] (see also the Detailed Considerations on Research Ethics section). One study noted the overrepresentation of affluent, highly educated White families among their participants and highlighted the need to target socioeconomically disadvantaged and racially diverse demographic groups with limited access to mental health resources [62]. Moreover, ensuring inclusivity for vulnerable groups was related to questions of access to digital technologies, in particular when developing interventions in the context of socioeconomically disadvantaged groups and regions [37,39,51] (see also the Accessibility section). One publication discussed how enabling users to snooze app notifications allowed them to use the intervention without fear of stigma [45].

In addition, the studies focused on unique aspects of vulnerability and highlighted the importance of reflections on decision-making processes in intervention development [32,34,37,39,44-46,60,62]. For instance, one study emphasized the need to tailor virtual reality interventions to the special needs of socially isolated adolescents in medical settings. It provided a discussion of technical and design features that alleviate motion sickness, a common problem during chemotherapy treatments [61].

Finally, the studies underscored the limitations of one-size-fits-all approaches. This aimed at ensuring the relevance and effectiveness of interventions for vulnerable populations, for example, in the context of Indigenous communities [32], and advocating for cultural adaptations (see the Cultural and Social Sensitivity section). A publication on a collaborative in-class intervention implicitly noted the risk of reinforcing differences between children less familiar with video games or slower at tasks and those who finish quickly. To prevent faster players from getting bored, it suggested offering them additional mini games until the others caught up [55]. One intervention aimed at children with social skill challenges reasoned to choose a single-player design to “create a safe environment in which to practice fledgling skills without social ramifications and avoid the possible iatrogenic effects of participants reinforcing negative behaviours in other children” [33]. Moreover, the studies reflected on the challenge of adapting digital interventions to “a span of ages, disorders, and abilities” [57] and to the personal needs of patients in the context of face-to-face therapy, in which personalization, through, for example, changing the order of levels, may “undermine the validated integrity of the intervention” [63].

### Social Implications

#### Overview

Approximately two-thirds (23/38, 61%) of the publications addressed social implications, including issues related to implementation in specific social and cultural contexts, relationships with other therapeutic options, economic aspects, and the social embeddedness of technologies in broader power dynamics. Moreover, only one publication reflected on the accreditation process for health technologies. Going through the process with a regional health quality agency encouraged ethical reflection through safety, accessibility, usability, and updating requirements. Feedback from this process prompted changes such as implementing a revision calendar and a user tool for suggestions to the app’s administrators [43].

#### Implementation Using Facilitators in Specific Social Contexts

Some publications (6/38, 16%) emphasized the importance of “a plan for real-world implementation” [41] that guarantees that young people actually use the intervention [41,52,61]. Suggested strategies included involving youth ambassadors via social media and manuals for facilitators such as teachers [41]. One intervention provided “guidelines about the resource’s good practices” [64] for participating youth upon registration and the possibility to report misconduct and disrespectful messages [64]. Often, the complementary use of digital interventions with face-to-face interactions was recommended, for example, advocating for a “blended facilitated approach” [44]. One study adapted an intervention for anger and aggression initially tested in a hospital to provide nonstigmatized care to children from minority groups and of lower socioeconomic statuses, addressing the challenge that “most therapies for children fail in community ‘real-world’ settings” [35].

Several publications (4/38, 11%) addressed the role of facilitators. For example, an intervention aimed at refugee youth emphasized the importance of facilitators to debrief “hard to deal with” [51] themes, for example, through a relaxation exercise. Another intervention in the sub-Saharan African context involved peer mentors (ie, trained lay workers) who actively reached out by phone on a weekly basis to improve program adherence and answer technical questions [37]. One publication recommended school-based interventions to be led by teachers instead of mental health professionals because of their preexisting connections with the adolescents [65]. In a video game for children aged 6 to 10 years, empowerment was engendered by facilitator-led group activities, in particular “group processes like respect, inclusion, sharing and belonging, which were transferrable to a small online group through sensitive and skilled facilitation” [44]. The same study thematized how facilitators’ initial hesitance toward digital mental health technologies was mitigated through intervention implementation, which resulted in increased “technical confidence and programme fidelity” [44]. Thereby, it shows how the use of technologies can increase acceptability.

Furthermore, the studies highlighted direct social interactions with persons experiencing mental disorders as beneficial and effective [30,53]. For instance, one study discussed how face-to-face interactions with “lived experience workers” [30] who had a history of depression fostered “an environment of reciprocity, making it easier for students to share their own stories” [30]. It also recommended that a trained teacher or, ideally, a mental health care professional should be present during these interactions to support students in need of assistance and familiarize them with existing mental health support offers in schools [30]. Direct social interactions were also found to reinforce destigmatizing effects, for example, when young people shared their intervention experiences with friends and family [51].

Parent and family involvement were discussed across several studies (4/38, 11%) [32,44,50,57]. One study focusing on Maori youth highlighted a “collectivist approach” [32], involving whanau (ie, family group) during both development and implementation, for example, providing resources and information to support children’s use of interventions [32]. Critically reflecting on parent involvement, other publications raised concerns, especially when families are involved in therapeutic difficulties or when parents are hesitant to support program participation [50]. In addition, one study noted positive effects of digital interventions on family dynamics, noting improvements in relationships and increased insights among family members [44].

Moreover, the studies highlighted the role of schools in successful implementation [43]. For example, it was recommended to integrate interventions into existing school-based prevention programs [48,52] and into the school curriculum to “increase normalization of mental health education at school” [30]. Two of the studies (2/38, 5%) addressed concerns regarding adolescent screen time with the active involvement of schools [37,46], for example, through “counsellor-supported use of smartphones during dedicated school-based sessions” [46].

Finally, one publication posited normative claims, which are statements about how things should be or what actions are considered right, regarding developers’ and researchers’ responsibilities after intervention development. It claimed that “researchers should accept the ongoing responsibility to gather data that helps to establish the boundaries of acceptable use and update and evolve guidelines accordingly” [50].

#### Relationship With Other Therapeutic Options

Several publications (4/38, 11%) emphasized the embeddedness of digital interventions within other therapeutic options, underscoring the ongoing importance of face-to-face therapy [44,50,54,62]. For instance, one study found that children generally preferred in-person interactions due to the intimacy and meaningfulness that arises from being physically together, the enjoyment of shared activities, and the ability to communicate openly. It highlighted “the potential for combined, engaging and resource-effective approaches” [44] that blend digital and face-to-face elements to cater to varying levels of needs effectively [44]. The studies framed digital interventions as “technology-enabled services, which serve to support the overall service or therapeutic process” [50], rather than as stand-alone solutions. In addition, the establishment of “communities of practice” [50] was suggested, where therapists and intervention developers could share positive and negative experiences with new technologies to prompt discussions about “how technologies fit with the broader ecosystem” [50]. For example, therapists encountered challenges when installing interventions on organizational computers and when needing parental consent for app installation [50].

Regarding therapists’ autonomy, there was acknowledgment of the diverse ways in which therapists use digital interventions [50]. Some therapists used digital tools as their primary approach, whereas others only turned to them when traditional face-to-face methods were met with resistance from young people [50]. One publication also mentioned the possibility to alternate between digital and other therapeutic elements within a single session. In terms of gamification elements, one approach involved implementing “a specific start and end” [54] to allow children to transition seamlessly from device use to other therapy activities.

#### Economic Aspects

Two publications (2/38, 11%) addressed the economic aspects of developing and implementing digital mental health interventions. One study advocated integrating cost-effective commercial digital interventions into clinical settings to address resource constraints and treatment delays at the health system level [57]. Moreover, one publication disclosed potential financial interests, indicating that the authors’ nonuniversity organization “may benefit financially from the sale of this game” [33].

Another topic was funding. The challenge of limited funding for serious games compared to commercial games was noted [40]. One publication stressed scientists’ responsibility to actively influence the commercial gaming industry, advocating for their proactive engagement to demonstrate “the financial, as well as health, benefits of providing beautiful, entertaining, and scientifically validated mental health tools” [52]. In a nonprofit intervention, funding limitations were a significant concern, requiring cofunding during development and additional funding after development for maintenance and updates to enhance user engagement [43].

#### Social Embeddedness of Technologies

The studies rarely reflected on the social embeddedness of technologies within larger governance and power mechanisms. One publication addressed concerns of adolescents at risk of school refusal regarding a gamified intervention being “yet another thing to deal with” [34] among the already overwhelming demands of their daily lives. To mitigate this concern, it was suggested to provide players with a “sense of mastery from their particular position” [34], offer positive feedback regardless of outcomes, and be mindful toward in-game formulations [34].

Another publication explicitly considered digital mental health interventions within larger power dynamics [31]. It found Indigenous youth’s reluctance to share personal information via an application being related to historical experiences of colonization. This reflects “the remaining ties between technology and colonization, which tend to position technology as having Western-European ontologies and the legacy of unethical research practices” [31].

## Discussion

### Principal Findings

From our review of 38 publications, we identified and classified various ethical aspects into four areas: (1) research ethics, (2) ethical principles (including privacy, accessibility, empowerment and autonomy, cultural and social sensitivity, co-design, and psychological and educational value), (3) vulnerable groups, and (4) social implications (including implementation using facilitators in specific social contexts, relationship with other therapeutic options, economic aspects, and social embeddedness of technologies). In the following sections, we highlight how our analysis shows instrumental conceptions of co-design and pragmatic approaches to vulnerability, a limited discussion of technologies’ social embeddedness in current research, and a limited interpretation of “ethics” as research ethics across the analyzed studies.

#### Instrumental Conceptions of Co-Design and Pragmatic Approaches to Vulnerability

While 18% (7/38) of the studies recognized the value of co-design, the rationale for adopting this approach often focused on enhancing appeal and acceptability rather than prioritizing inclusivity, fostering multiperspective reflection, or engaging in other ethical considerations. The risk here is reducing co-design to a tool for determining the preferences of particular groups of young people. In contrast, research on co-design in technology development has advocated for “the development of co-design methodologies that include ethical issues in more explicit and comprehensive ways” [68]. These research approaches shift the focus to shared responsibilities among developers, users, and the public.

Studies focusing on vulnerable groups seldom discussed intersectionality, referring to the compounded impact of multiple disadvantages. Instead, these studies addressed the mental health needs of vulnerable groups pragmatically, aiming to enable the design and implementation of effective interventions for the targeted group. Moreover, while the papers emphasized the importance of addressing the needs of the specific vulnerable group under study, they lacked reflections on the decision-making process for prioritizing one vulnerable group over others. In addition, some studies highlighted the tension between personalized mental health services and one-size-fits-all digital interventions that may not cater to individual needs, especially when personally experiencing vulnerability. While customization options such as customizable avatars can address this issue by enhancing cultural and social sensitivity, therapists in particular warned about the potential drawbacks compared to personalized care practices.

#### Limited Discussion of the Social Embeddedness of Technologies

Social constructivists have established that the meaning and use of technology can only be understood by taking its social embeddedness into consideration [69]. Technology and society are interdependent, mutually shaping each other [70]—social factors shape the purpose, methods, and objectives of technology design, whereas technology may facilitate social change. The studies included in this review rarely discussed this interdependency. An exception was a minor comment in one publication, which addressed how facilitators’ initial doubts about digital mental health tools were overcome during intervention implementation [44]. These findings not only demonstrate how implementing a digital intervention can enhance acceptance but also implicitly address how it ultimately alters people’s perceptions of this technology.

Moreover, only 2 of the publications (2/38, 5%) discussed the social embeddedness of technologies within larger governance and power mechanisms. One study on developing an intervention for Indigenous young people addressed the risk of taking up colonial legacies [31], and another publication reflected on how digital mental health interventions might reproduce the structural burden of work overload and stress [34]. This relates to critical scholarship on serious games, which discusses the use of serious games for increasing mental health as part of neoliberal governance strategies, which control life by applying metrics of utility, productivity, and competitiveness [71].

In addition, future research should further explore how accreditation processes with governmental health agencies can influence ethically relevant design choices, which was only mentioned in the study by Duarte-Hueros et al [43]. In addition, insights on decisions not to seek accreditation for health technologies would be valuable. In fact, almost all the publications on gamified digital mental health technologies for young people either omitted or did not mention undergoing these regulatory procedures.

Finally, most publications (37/38, 97%) largely overlooked environmental factors except for one study that tentatively claimed environmental friendliness due to reduced paper use compared to traditional therapeutic methods [49]. However, as part of reflecting accessibility issues, other publications implicitly discussed environmental considerations, such as addressing limited storage space and internet access in socioeconomically disadvantaged areas. However, it is also crucial to reflect on data storage and server load generated by digital interventions in more affluent contexts.

#### Identifying Ethical Aspects Beyond Research Ethics

When excluding 43 studies at the full-text screening stage, we were surprised by how many publications did not report on “ethics” at all (n=8, 21%), only mentioned an obtained ethics committee vote (n=10, 26%), or reported on ethically relevant decisions without discussing them (n=9, 24%; Figure 1). While we cannot assert whether or how the broader projects underlying these 23 publications addressed ethical considerations elsewhere or through their practices, our review indicates a scarcity of ethical reflection in publications concerning gamified digital mental health interventions for young people. A considerable proportion of studies in this review (21/38, 55%) centered on user experiences. This is unsurprising given our inclusion criteria limited to studies addressing ethical aspects. Qualitative methodologies in particular tend to foreground ethical considerations, facilitating the emergence of novel themes and fostering critical reflection among users and their care providers regarding the intervention.

Our results show reflections on diverse ethical challenges and advantages. However, the studies rarely framed these reflections as addressing ethical aspects. Hence, the terms “ethics” and “ethical” were only used in the context of research ethics across the 38 included publications. This indicates an overall narrow view of ethics, which confines ethics to aspects relevant only to interactions with research participants, potentially relegating it to a bothersome and technocratic prerequisite for institutional approval. This risks missing out on important reflections on ethical aspects, which are pertinent not only to research participant involvement during usability testing and effectiveness measurements but also across the entire technology development cycle. One exception is a short conference paper on an intervention with wearable biosensor technology and 3D holographic displays. It explicitly addressed “ethical challenges” [66] in the form of raising questions without providing further discussions. It asked the following: “Can a robotic agent manage or mitigate emotional or empathetic distress in young children? What are the negative consequences of enabling children to interact with each other’s e-worlds? How do children feel about sharing their memories in a public space?” [66].

Concerning research ethical reflection, while 82% (31/38) of the studies indicated an obtained research ethics committee vote, only 26% (10/38) provided detailed considerations of research ethics. These insights align with an ongoing scholarly debate on the drawbacks of the expansion of ethics committees and similar institutions. Social scientists warn of the potential disconnect between adhering to predefined ethical codes of conduct in scholarly research stemming from these developments [72-74]. This review shows how ethical reflections can help address critical decisions at every step of the technology development process.

### Limitations and Future Research

Several limitations as well as avenues for future research emerge. First, during the screening process, contrary to our initial expectations, it was not possible to assess the relevance of publications regarding ethical aspects based solely on titles and abstracts. Consequently, we opted to set aside ethics-related inclusion and exclusion criteria during abstract and full-text screening, considering them only during full-text assessment. While this merits further discussion on how the inclusion of ethics-related terms shape the search, one limitation of our methodology is that we did not cross-verify the relevance of studies excluded by our ethics-related search terms.

Second, we included relevant gray literature only in our discussion due to feasibility constraints, but future studies could explore it more thoroughly for potentially interesting results. Third, this review examined interventions targeting various mental health aspects across the board. In future research, it would be beneficial to address the specific ethical considerations tied to different understandings of mental health. For instance, interventions aimed at enhancing social connections may encounter distinct ethical challenges compared to those focused on raising awareness about depressive symptoms. Fourth, the disciplinary backgrounds of the study authors might deserve closer attention in future research as they might potentially foster discussions and negotiations of conflicting values.

Fifth, an existing ethical framework for gamified fitness-tracking apps distinguishes among ethical considerations related to design, use, and embeddedness in the broader social context [75]. Our findings implicitly reflect these distinctions. Future research could further clarify how ethical issues distinctly manifest at different project stages of gamified digital mental health intervention development and implementation. Moreover, the reviewed publications did not address common ethical concerns in the literature on ethical issues in gamification, such as exploitation, manipulation, competition, or addiction, which have been raised in other contexts [76] such as corporate work [77]. This omission may be due to the publications’ focus not being on ethics, or it could reflect the underexplored area of mental health in gamification. This yields opportunities to further examine the unique ethical issues within the mental health context.

Seventh, although we initially aimed to analyze ethical advantages, the publications primarily mentioned these as part of the interventions’ general context. They referenced the work of others rather than providing concrete insights from their specific intervention developments, such as health economic calculations on the actual cost-effectiveness of a particular intervention. Such discussions tend to formulate overloaded expectations without an empirical basis. This aspect deserves closer attention to mitigate the risk of bordering on rhetoric and merely invoking “solutionism”—the belief that technology can solve all problems [78]. Thereby, digital “solutions” might hinder more holistic approaches and changes to structural flaws.

Finally, only 16% (6/38) of the reviewed publications addressed interventions in Asia, Africa, and South America. Of these, only Uganda and India are classified by the World Bank as low- or lower-middle-income countries. Future research on digital mental health interventions for young people should further highlight the importance of understanding the ethical challenges in these areas of the world [79].

### Conclusions

The aim of this scoping review was to map the ethical aspects of developing and implementing gamified digital mental health interventions for young people. It showed how the 38 publications included for analysis discussed ethical aspects across the areas of research ethics, ethical principles, vulnerable groups, and social implications. It provided concrete examples from real-world intervention development. Thereby, our findings illustrate how ethical issues manifest in different interventions and (do not) prompt diverse reflections, mitigation strategies, and actions. From this perspective, ethics can be seen as an ongoing practice not only in research ethical considerations or in self-imposed “ethics checklists” but also across all project stages. Examples include economic considerations on funding, which become pertinent already at the very onset of a project, and the involvement of facilitators such as teachers and therapists during development but also afterward when implementing an intervention.

Methodologically, this review used an abductive approach [29] to ethics informed by existing research and guidelines on digital mental health interventions, our own practical knowledge as ethics researchers, and sensitivity toward empirically emerging ethical aspects from the included publications. By not working with a predefined and closed conception of ethics, we were able to effectively identify ethically significant decisions and considerations that were not explicitly labeled as ethical reflections in the publications selected for review.

To conclude, while existing research has rarely addressed the ethical aspects of gamified digital mental health interventions for adolescents explicitly, this review identified and analyzed how publications have addressed ethically relevant decisions and considerations involved in developing and implementing these interventions. The examples of ethical reflections provided in this paper should not be taken as “good solutions” in the sense of best-practice examples. Rather, this review maps the breadth of ethical discussions, aiming to foster an understanding of serious game ethics as an ongoing practice across all project stages. Beyond ethics checklists, this review advocates for collaborative critical reflection among mental health researchers and game developers.

## Appendix

Multimedia Appendix 1 PRISMA-ScR (Preferred Reporting Items for Systematic reviews and Meta-Analyses extension for Scoping Reviews) checklist.

Multimedia Appendix 2 Search terms and search strings for each database.

Multimedia Appendix 3 Table with all ethics-related direct quotes and summaries extracted from the 38 included publications.

Multimedia Appendix 4 List of the key characteristics of the 38 included publications.

## Abbreviations

RQ: research question
