# Supplementary material for: Gamified Digital Mental Health Interventions for Young People: Scoping Review of Ethical Aspects During Development and Implementation
Source: JMIR Serious Games. 2024 Nov 28;12:e64488. doi: 10.2196/64488 (PMC11638686; doi:10.2196/64488)
Supplement: Multimedia Appendix 2 [file games_v12i1e64488_app2.docx]

**List of search terms**

| *Search group* | *Search terms* |
| --- | --- |
| **Outcome: ethical challenges and advantages** | ethics ethical moral value* ELSI ELSA risk* benefit* potential challenge* autonomy empower* privacy confidentiality trust consent stigma responsibility "regulatory framework*" safety accessibility equity inequity equality inequality bias "digital literacy" socio-economic "social determinant*" exclusion |
| **Intervention: gamified** | gamified gamification game* gaming videogame |
| **Intervention: digital** | digital digitised virtual VR computer* "augmented realit*" AR e-health app apps application* "mobile technolog*" "mobile device*" smartphone* phone* "handheld device*" interactive "digital device*" m-health mhealth |
| **Intervention: mental health** | "mental health" wellbeing well-being "mental illness*" "mental disorder*" belonging affiliation* "social connection*" relatedness vulnerab* |
| **Population: adolescents** | adolescent* adolescence teenager* child* youth student* |

**Search strings for each database**

| *Database* |  | *Search string* |
| --- | --- | --- |
| **Scopus** |  | ( ( TITLE-ABS-KEY ( ethics ) OR TITLE-ABS-KEY ( ethical ) OR TITLE-ABS-KEY ( moral ) OR TITLE-ABS-KEY ( value* ) OR TITLE-ABS-KEY ( elsi ) OR TITLE-ABS-KEY ( elsa ) OR TITLE-ABS-KEY ( risk* ) OR TITLE-ABS-KEY ( benefit* ) OR TITLE-ABS-KEY ( potential ) OR TITLE-ABS-KEY ( challenge* ) OR TITLE-ABS-KEY ( autonomy ) OR TITLE-ABS-KEY ( empower* ) OR TITLE-ABS-KEY ( privacy ) OR TITLE-ABS-KEY ( confidentiality ) OR TITLE-ABS-KEY ( trust ) OR TITLE-ABS-KEY ( consent ) OR TITLE-ABS-KEY ( stigma ) OR TITLE-ABS-KEY ( responsibility ) OR TITLE-ABS-KEY ( "regulatory framework*" ) OR TITLE-ABS-KEY ( safety ) OR TITLE-ABS-KEY ( accessibility ) OR TITLE-ABS-KEY ( equity ) OR TITLE-ABS-KEY ( inequity ) OR TITLE-ABS-KEY ( equality ) OR TITLE-ABS-KEY ( inequality ) OR TITLE-ABS-KEY ( bias ) OR TITLE-ABS-KEY ( "digital literacy" ) OR TITLE-ABS-KEY ( socio-economic ) OR TITLE-ABS-KEY ( "social determinant*" ) OR TITLE-ABS-KEY ( exclusion ) ) AND ( TITLE-ABS-KEY ( gamified ) OR TITLE-ABS-KEY ( gamification ) OR TITLE-ABS-KEY ( game* ) OR TITLE-ABS-KEY ( gaming ) OR TITLE-ABS-KEY ( videogame ) ) AND ( TITLE-ABS-KEY ( digital ) OR TITLE-ABS-KEY ( digitised ) OR TITLE-ABS-KEY ( virtual ) OR TITLE-ABS-KEY ( vr ) OR TITLE-ABS-KEY ( computer* ) OR TITLE-ABS-KEY ( "augmented realit*" ) OR TITLE-ABS-KEY ( ar ) OR TITLE-ABS-KEY ( e-health ) OR TITLE-ABS-KEY ( app ) OR ( apps ) OR TITLE-ABS-KEY ( application* ) OR TITLE-ABS-KEY ( "mobile technolog*" ) OR TITLE-ABS-KEY ( "mobile device*" ) OR TITLE-ABS-KEY ( smartphone* ) OR TITLE-ABS-KEY ( phone* ) OR TITLE-ABS-KEY ( "handheld device*" ) OR TITLE-ABS-KEY ( "digital device*" ) OR TITLE-ABS-KEY ( interactive ) OR TITLE-ABS-KEY ( m-health ) OR TITLE-ABS-KEY ( mhealth ) ) AND ( TITLE-ABS-KEY ( "mental health" ) OR TITLE-ABS-KEY ( wellbeing ) OR TITLE-ABS-KEY ( well-being ) OR TITLE-ABS-KEY ( "mental illness*" ) OR TITLE-ABS-KEY ( "mental disorder*" ) OR TITLE-ABS-KEY ( belonging ) OR TITLE-ABS-KEY ( affiliation* ) OR TITLE-ABS-KEY ( "social connection*" ) OR TITLE-ABS-KEY ( relatedness ) OR TITLE-ABS-KEY ( vulnerab* ) ) AND ( TITLE-ABS-KEY ( adolescent* ) OR TITLE-ABS-KEY ( adolescence ) OR TITLE-ABS-KEY ( teenager* ) OR TITLE-ABS-KEY ( child* ) OR TITLE-ABS-KEY ( youth ) OR TITLE-ABS-KEY ( student* ) ) ) AND PUBYEAR > 2014 AND PUBYEAR < 2024 AND ( LIMIT-TO ( DOCTYPE,"ar" ) OR LIMIT-TO ( DOCTYPE,"cp" ) ) AND ( LIMIT-TO ( LANGUAGE,"English" ) OR LIMIT-TO ( LANGUAGE,"Spanish" ) OR LIMIT-TO ( LANGUAGE,"German" ) ) |
| **Web of Science** | S1 | TI=(ethics) OR TI=(ethical) OR TI=(moral) OR TI=(value*) OR TI=(ELSI) OR TI=(ELSA) OR TI=(risk*) OR TI=(benefit*) OR TI=(potential) OR TI=(challenge*) OR TI=(autonomy) OR TI=(empower*) OR TI=(privacy) OR TI=(confidentiality) OR TI=(trust) OR TI=(consent) OR TI=(stigma) OR TI=(responsibility) OR TI=("regulatory framework*") OR TI=(safety) OR TI=(accessibility) OR TI=(equity) OR TI=(inequity) OR TI=(equality) OR TI=(inequality) OR TI=(bias) OR TI=("digital literacy") OR TI=(socio-economic) OR TI=("social determinant*") OR TI=(exclusion) OR AB=(ethics) OR AB=(ethical) OR AB=(moral) OR AB=(value*) OR AB=(ELSI) OR AB=(ELSA) OR AB=(risk*) OR AB=(benefit*) OR AB=(potential) OR AB=(challenge*) OR AB=(autonomy) OR AB=(empower*) OR AB=(privacy) OR AB=(confidentiality) OR AB=(trust) OR AB=(consent) OR AB=(stigma) OR AB=(responsibility) OR AB=("regulatory framework*") OR AB=(safety) OR AB=(accessibility) OR AB=(equity) OR AB=(inequity) OR AB=(equality) OR AB=(inequality) OR AB=(bias) OR AB=("digital literacy") OR AB=(socio-economic) OR AB=("social determinant*") OR AB=(exclusion) OR AK=(ethics) OR AK=(ethical) OR AK=(moral) OR AK=(value*) OR AK=(ELSI) OR AK=(ELSA) OR AK=(risk*) OR AK=(benefit*) OR AK=(potential) OR AK=(challenge*) OR AK=(autonomy) OR AK=(empower*) OR AK=(privacy) OR AK=(confidentiality) OR AK=(trust) OR AK=(consent) OR AK=(stigma) OR AK=(responsibility) OR AK=("regulatory framework*") OR AK=(safety) OR AK=(accessibility) OR AK=(equity) OR AK=(inequity) OR AK=(equality) OR AK=(inequality) OR AK=(bias) OR AK=("digital literacy") OR AK=(socio-economic) OR AK=("social determinant*") OR AK=(exclusion) |
|  | S2 | TI=(gamified) OR TI=(gamification) OR TI=(game*) OR TI=(gaming) OR TI=(videogame) OR AB=(gamified) OR AB=(gamification) OR AB=(game*) OR AB=(gaming) OR AB=(videogame) OR AK=(gamified) OR AK=(gamification) OR AK=(game*) OR AK=(gaming) OR AK=(videogame) |
|  | S3 | TI=(digital) OR TI=(digitised) OR TI=(virtual) OR TI=(VR) OR TI=(computer*) OR TI=("augmented realit*") OR TI=(AR) OR TI=(e-health) OR TI=(app) OR TI=(apps) OR TI=(application*) OR TI=("mobile technolog*") OR TI=("mobile device*") OR TI=(smartphone*) OR TI=(phone*) OR TI=("handheld device*") OR TI=(interactive) OR TI=("digital device*") OR TI=(m-health) OR TI=(mhealth) OR AB=(digital) OR AB=(digitised) OR AB=(virtual) OR AB=(VR) OR AB=(computer*) OR AB=("augmented realit*") OR AB=(AR) OR AB=(e-health) OR AB=(app) OR AB=(apps) OR AB=(application*) OR AB=("mobile technolog*") OR AB=("mobile device*") OR AB=(smartphone*) OR AB=(phone*) OR AB=("handheld device*") OR AB=(interactive) OR AB=("digital device*") OR AB=(m-health) OR AB=(mhealth) OR AK=(digital) OR AK=(digitised) OR AK=(virtual) OR AK=(VR) OR AK=(computer*) OR AK=("augmented realit*") OR AK=(AR) OR AK=(e-health) OR AK=(app) OR AK=(apps) OR AK=(application*) OR AK=("mobile technolog*") OR AK=("mobile device*") OR AK=(smartphone*) OR AK=(phone*) OR AK=("handheld device*") OR AK=(interactive) OR AK=("digital device*") OR AK=(m-health) OR AK=(mhealth) |
|  | S4 | TI=("mental health") OR TI=(wellbeing) OR TI=(well-being) OR TI=("mental illness*") OR TI=("mental disorder*") OR TI=(belonging) OR TI=(affiliation*) OR TI=("social connection*") OR TI=(relatedness) OR TI=(vulnerab*) OR AB=("mental health") OR AB=(wellbeing) OR AB=(well-being) OR AB=("mental illness*") OR AB=("mental disorder*") OR AB=(belonging) OR AB=(affiliation*) OR AB=("social connection*") OR AB=(relatedness) OR AB=(vulnerab*) OR AK=("mental health") OR AK=(wellbeing) OR AK=(well-being) OR AK=("mental illness*") OR AK=("mental disorder*") OR AK=(belonging) OR AK=(affiliation*) OR AK=("social connection*") OR AK=(relatedness) OR AK=(vulnerab*) |
|  | S5 | TI=(adolescent*) OR TI=(adolescence) OR TI=(teenager*) OR TI=(child*) OR TI=(youth) OR TI=(student*) OR AB=(adolescent*) OR AB=(adolescence) OR AB=(teenager*) OR AB=(child*) OR AB=(youth) OR AB=(student*) OR AK=(adolescent*) OR AK=(adolescence) OR AK=(teenager*) OR AK=(child*) OR AK=(youth) OR AK=(student*) |
|  | S6 | S1 AND S2 AND S3 AND S4 AND S5 (limited to 2015-2023 and to the document types “article”, “proceeding paper”, and “early access”) |
| **MEDLINE**: Publisher, In-Data-Review, In-Process and PubMed-not-MEDLINE | 1 | (ethics or ethical or moral or value* or ELSI or ELSA or risk* or benefit* or potential or challenge* or autonomy or empower* or privacy or confidentiality or trust or consent or stigma or responsibility or "regulatory framework*" or safety or accessibility or equity or inequity or equality or inequality or bias or "digital literacy" or socio-economic or "social determinant*" or exclusion).ti. or (ethics or ethical or moral or value* or ELSI or ELSA or risk* or benefit* or potential or challenge* or autonomy or empower* or privacy or confidentiality or trust or consent or stigma or responsibility or "regulatory framework*" or safety or accessibility or equity or inequity or equality or inequality or bias or "digital literacy" or socio-economic or "social determinant*" or exclusion).ab. or (ethics or ethical or moral or value* or ELSI or ELSA or risk* or benefit* or potential or challenge* or autonomy or empower* or privacy or confidentiality or trust or consent or stigma or responsibility or "regulatory framework*" or safety or accessibility or equity or inequity or equality or inequality or bias or "digital literacy" or socio-economic or "social determinant*" or exclusion).kw. or bioethical issues/ or bioethics/ or ethical analysis/ or "ethical review"/ or ethicists/ or principle-based ethics/ or health equity/ or healthcare disparities/ or Digital Divide/ or health inequities/ or socioeconomic disparities in health/ or "Social Determinants of Health"/ or health status disparities/ |
|  | 2 | (gamified or gamification or game* or gaming or videogame).ti. or (gamified or gamification or game* or gaming or videogame).ab. or (gamified or gamification or game* or gaming or videogame).kw. or gamification/ or "Play and Playthings"/ |
|  | 3 | (digital or digitised or virtual or VR or computer* or "augmented realit*" or AR or e-health or app or apps or application* or "mobile technolog*" or "mobile device*" or smartphone* or phone* or "handheld device*" or interactive or "digital device*" or m-health or mhealth).ti. or (digital or digitised or virtual or VR or computer* or "augmented realit*" or AR or e-health or app or apps or application* or "mobile technolog*" or "mobile device*" or smartphone* or phone* or "handheld device*" or interactive or "digital device*" or m-health or mhealth).ab. or (digital or digitised or virtual or VR or computer* or "augmented realit*" or AR or e-health or app or apps or application* or "mobile technolog*" or "mobile device*" or smartphone* or phone* or "handheld device*" or interactive or "digital device*" or m-health or mhealth).kw. or Digital Technology/ or augmented reality/ or virtual reality/ or user-centered design/ or user-computer interface/ or Mobile Applications/ or computers, handheld/ or smartphone/ |
|  | 4 | ("mental health" or wellbeing or well-being or "mental illness*" or "mental disorder*" or belonging or affiliation* or "social connection*" or relatedness or vulnerab*).ti. or ("mental health" or wellbeing or well-being or "mental illness*" or "mental disorder*" or belonging or affiliation* or "social connection*" or relatedness or vulnerab*).ab. or ("mental health" or wellbeing or well-being or "mental illness*" or "mental disorder*" or belonging or affiliation* or "social connection*" or relatedness or vulnerab*).kw. or Mental Health/ or Mental Disorders/ or social vulnerability/ |
|  | 5 | (adolescent* or adolescence or teenager* or child* or youth or student*).ti. or (adolescent* or adolescence or teenager* or child* or youth or student*).ab. or (adolescent* or adolescence or teenager* or child* or youth or student*).kw. or adolescent/ or young adult/ or child/ or Students/ |
|  | 6 | 1 and 2 and 3 and 4 and 5 and 2015:2023.(sa_year). |
| **PsycINFO** | 1 | (ethics or ethical or moral or value* or ELSI or ELSA or risk* or benefit* or potential or challenge* or autonomy or empower* or privacy or confidentiality or trust or consent or stigma or responsibility or "regulatory framework*" or safety or accessibility or equity or inequity or equality or inequality or bias or "digital literacy" or socio-economic or "social determinant*" or exclusion).ti. or (ethics or ethical or moral or value* or ELSI or ELSA or risk* or benefit* or potential or challenge* or autonomy or empower* or privacy or confidentiality or trust or consent or stigma or responsibility or "regulatory framework*" or safety or accessibility or equity or inequity or equality or inequality or bias or "digital literacy" or socio-economic or "social determinant*" or exclusion).ab. or (ethics or ethical or moral or value* or ELSI or ELSA or risk* or benefit* or potential or challenge* or autonomy or empower* or privacy or confidentiality or trust or consent or stigma or responsibility or "regulatory framework*" or safety or accessibility or equity or inequity or equality or inequality or bias or "digital literacy" or socio-economic or "social determinant*" or exclusion).id. or bioethical issues/ or bioethics/ or ethical analysis/ or "ethical review"/ or ethicists/ or principle-based ethics/ or health equity/ or healthcare disparities/ or Digital Divide/ or health inequities/ or socioeconomic disparities in health/ or "Social Determinants of Health"/ or health status disparities/ |
|  | 2 | (gamified or gamification or game* or gaming or videogame).ti. or (gamified or gamification or game* or gaming or videogame).ab. or (gamified or gamification or game* or gaming or videogame).id. or gamification/ or "Play and Playthings"/ |
|  | 3 | (digital or digitised or virtual or VR or computer* or "augmented realit*" or AR or e-health or app or apps or application* or "mobile technolog*" or "mobile device*" or smartphone* or phone* or "handheld device*" or interactive or "digital device*" or m-health or mhealth).ti. or (digital or digitised or virtual or VR or computer* or "augmented realit*" or AR or e-health or app or apps or application* or "mobile technolog*" or "mobile device*" or smartphone* or phone* or "handheld device*" or interactive or "digital device*" or m-health or mhealth).ab. or (digital or digitised or virtual or VR or computer* or "augmented realit*" or AR or e-health or app or apps or application* or "mobile technolog*" or "mobile device*" or smartphone* or phone* or "handheld device*" or interactive or "digital device*" or m-health or mhealth).id. or Digital Technology/ or augmented reality/ or virtual reality/ or user-centered design/ or user-computer interface/ or Mobile Applications/ or computers, handheld/ or smartphone/ |
|  | 4 | ("mental health" or wellbeing or well-being or "mental illness*" or "mental disorder*" or belonging or affiliation* or "social connection*" or relatedness or vulnerab*).ti. or ("mental health" or wellbeing or well-being or "mental illness*" or "mental disorder*" or belonging or affiliation* or "social connection*" or relatedness or vulnerab*).ab. or ("mental health" or wellbeing or well-being or "mental illness*" or "mental disorder*" or belonging or affiliation* or "social connection*" or relatedness or vulnerab*).id. or Mental Health/ or Mental Disorders/ or social vulnerability/ |
|  | 5 | (adolescent* or adolescence or teenager* or child* or youth or student*).ti. or (adolescent* or adolescence or teenager* or child* or youth or student*).ab. or (adolescent* or adolescence or teenager* or child* or youth or student*).id. or adolescent/ or young adult/ or child/ or Students/ |
|  | 6 | 1 and 2 and 3 and 4 and 5 |
|  | 7 | limit 6 to ((english or german or spanish) and yr="2015 - 2023") |

### 
